# The Role of Mannose-Binding Lectin Serum Level in Tubotympanic Chronic Suppurative Otitis Media

**DOI:** 10.1155/2018/6178159

**Published:** 2018-05-22

**Authors:** Anton Budhi Darmawan, Marsetyawan H. N. E. Soesatyo, Ratna Dwi Restuti, Agus Surono

**Affiliations:** ^1^Department of Otorhinolaryngology, Head and Neck Surgery, Faculty of Medicine, Universitas Jenderal Soedirman-Margono Soekarjo Hospital, Purwokerto, Indonesia; ^2^Department of Cell Biology and Histology, Faculty of Medicine, Universitas Gadjah Mada, Yogyakarta, Indonesia; ^3^Department of Otorhinolaryngology, Head and Neck Surgery, Faculty of Medicine, Universitas Indonesia-Cipto Mangunkusumo Hospital, Jakarta, Indonesia; ^4^Department of Otorhinolaryngology, Head and Neck Surgery, Faculty of Medicine, Universitas Gadjah Mada-Sardjito Hospital, Yogyakarta, Indonesia

## Abstract

**Background:**

Chronic suppurative otitis media (CSOM) is a common public health problem worldwide and a major cause of hearing impairment especially in developing countries. The role of Mannose-Binding Lectin (MBL), a component of innate immunity, in CSOM has not been studied. The aim of the study was to examine whether MBL deficiency was more frequently present in cases group of tubotympanic CSOM patients rather than healthy subjects.

**Material and Methods:**

This was an analytic observational study. Subjects were enrolled in the Otorhinolaryngology Clinic at Margono Soekarjo Hospital, Purwokerto, Indonesia. An independent *t*-test was used to compare the mean of MBL serum concentration between tubotympanic CSOM subjects and control.

**Results:**

From 36 tubotympanic CSOM patients, there were 8 (22.22%) patients with MBL deficiency (MBL level < 100 ng/ml), while no deficiency was found in the control group. The mean of MBL level in cases group was 354.88 ng/ml, with the lowest level being 0.001 ng/ml and the highest level 690.24 ng/ml, while in the control group MBL level mean was 376.27 with the lowest level being 188.71 and the highest level 794.54 ng/ml.

**Conclusion:**

There was no significant difference of MBL serum level between tubotympanic CSOM and control group. However, the presence of subjects with MBL deficiency in the tubotympanic CSOM group might be considered as playing a role in the tubotympanic CSOM.

## 1. Introduction

Chronic suppurative otitis media (CSOM) is defined as a persistent discharge from the middle ear through a tympanic membrane perforation for more than 6 weeks. CSOM may include a chronic perforation with or without acute or chronic otorrhoea [1]. Traditionally, CSOM is classified into two types which are tubotympanic and atticoantral disease [2, 3]. Tubotympanic chronic otitis media tends to be typified by disease of the pars tensa and mesotympanum, whereas atticoantral disease primarily involves the pars flaccida and epitympanum [3]. CSOM is a common public health problem worldwide and a major cause of hearing impairment especially in developing countries [4]. The burden of CSOM varies worldwide. Global incidence rate of CSOM was 4.76 per thousand people for a total of 31 million cases [5]. Global prevalence rates are approximately between 1% and 46%; it has been estimated that 65–330 million individuals have discharging ears, 60% of whom suffered from significant hearing impairment. According to the World Health Organization (WHO), Western Pacific countries have the highest prevalence (2.5% to 43%), followed by South East Asia (0.9% to 7.8%), Africa (0.4% to 4.2%), South and Central America (3%), the Eastern Mediterranean (1.4%), and finally Europe (average prevalence of 0.4%) [4].

The occurrence of tubotympanic CSOM is caused by multiple factors such as viral or bacterial infections, tubal dysfunction, allergies, and immunological, environmental, and socioeconomic factors, but bacterial infections that enter the middle ear through nasopharynx was believed to be the most common cause [6–8]. In addition, bacterial infections also play an important role in the chronicity and recurrence of otitis media. One of the immune systems that play a role in eliminating bacteria is innate immune system [9]. The innate immune response provides an important defense in early life since passive maternal antibodies are reduced and there is a relative immaturity of the adaptive immune response [10]. Unlike the adaptive immune process which is pathogen-specific, the innate immune system provides a nonspecific defense mechanism, which also stimulates and modulates adaptive immune responses [11]. It uses pattern recognition receptors (PRRs) to recognize the molecular signature of pathogens, known as the pathogen-associated molecular patterns (PAMPs), to detect microbial infection [9, 11].

The actions of the innate immune system and the crosstalk with the slower but a longer-term adaptive immune system allow pathogens clearance, recovery of mucosal homeostasis, and prevention of persistent inflammation. Failure to recover homeostasis is thought to result in chronic inflammation [12].

One of the important molecules in the innate immune system that is involved in the first defense mechanism before antibody production is Mannose-Binding Lectin (MBL) [13]. MBL is a water-soluble protein, synthesized by hepatocyte cells in the liver. MBL binds not only carbohydrate groups to bacteria, but also phospholipids, nucleic acids, and nonglycosylase proteins, properties which may be relevant to apoptotic cell clearance [9].

Low level of MBL can cause a poor directed immune activity against several types of pathogens, and these incorrect reactions will affect many series events that will trigger the formation of polyps [14]. The aim of the current study was to compare the mean of MBL serum level in tubotympanic CSOM patients and in healthy subjects and moreover to examine whether MBL deficiency was more frequently present in a group of tubotympanic CSOM patients than in healthy subjects.

## 2. Material and Methods

This was an analytic observational study. Subjects were enrolled in the Otorhinolaryngology Clinic at Margono Soekarjo Hospital, Purwokerto, Indonesia. This study was approved by Research Ethics Committee of Universitas Jenderal Soedirman, Purwokerto, Indonesia, and informed consent was obtained from the subjects. The study consisted of 36 subjects of case group who had been diagnosed with tubotympanic CSOM and 36 subjects in healthy control group between the ages of 17 and 50 years.

Diagnostic criteria of tubotympanic CSOM based on clinical practice guidelines of the Indonesian Association of Otorhinolaryngology (PERHATI) are as follows: (1) persistent or recurrence of ear discharge for more than two months; (2) perforated tympanic membrane; (3) negative findings of cholesteatoma from physical examination or radiological examination [15].

Subjects who had suffered from infection of the external ear such as diffuse external otitis or otomycosis and subjects who had history of HIV-AIDS, diabetes mellitus, and autoimmune disease were excluded from this study. Subjects who were taking steroid medicine and subjects who did not cooperate with the examination were also excluded. The serum MBL levels of patients and controls were measured by sandwich enzyme immunoassay using a Human MBL ELISA Kit (Bioporto®, Denmark). Diluted samples and standards were incubated in microtiter wells coated with monoclonal antibody mannan at room temperature for 1 hour. After washing, the plates were incubated for 1 hour with diluted tracer, followed by incubation for 1 hour with diluted streptavidin–peroxidase conjugate. Color was developed using tetramethylbenzidine (TMB) substrate solution, and the reaction was stopped by adding stop solution. The plates were placed in a spectrophotometer and the absorbance was measured at 450 nm. An independent *t*-test was used to compare the mean of MBL serum concentration between tubotympanic CSOM subjects and control.

## 3. Results

Out of 36 cases studied, 11 (30.5%) were male and 25 (69.5%) female. The average age was 37.29 years with range of 17–60 years old. In the control group, there were 17 (47.2%) male and 19 (52.8%) female. The average age was 30.42 years with minimum age was 17 and maximum age was 52 years old (Table 1). From 36 cases studied, there were 47 ears suffering from tubotympanic CSOM, of which 25 (69.4%) were bilateral and 11 (30.6%) unilateral. Thirty-four ears (72.3%) were active condition, while 13 (27.7%) ears were inactive.

The mean of MBL level in cases group was 354,88 ng/ml, with the lowest level being 0.001 ng/ml and the highest level 690.24 ng/ml, while in the control group MBL level mean was 376.27 with the lowest level being 188.71 and the highest level 794.54 ng/ml. From 36 tubotympanic CSOM patients, we found 8 (22.22%) patients with MBL deficiency (MBL level < 100 ng/ml [16]), while no deficiency was found in the control group.

Independent *t*-test showed that there was no significant difference of MBL serum level between tubotympanic CSOM and control group (*p* > 0.05).

## 4. Discussion

Several studies that elaborate the role of MBL in health and disease have been published. In the otorhinolaryngology field, research on MBL relationship with some diseases has been done with varying results, such as the research conducted by Homøe et al. which shows that there was no significant relationship between MBL genotype and otitis media. On the other hand, a research conducted by Nuytinck et al. suggests that there was a meaningful relationship between MBL genotype with recurrent acute otitis media [9, 17]. However, there were limitations of this study to demonstrate the role of MBL to CSOM, one of which is the relatively small number of samples.

The mean of MBL level of the tubotympanic CSOM group was 354.88 ng/ml, whereas the mean MBL concentration of the control group in this study was 376.27 ng/ml. These data indicate that the mean MBL levels of both groups were within normal limits, since MBL deficiency is defined as an MBL serum concentration below 100 ng/mL [16]. Although there was no statistically significant difference in MBL serum levels between tubotympanic CSOM patients and healthy subjects, the study found that 8 (22.2%) patients had MBL deficiency, while no deficiency was found in the control group. We found previous literature which suggest that MBL deficiency is associated with chronicity of disease, namely, hepatitis C [18] and chronic periodontitis [19]. This suggests that MBL deficiency may be clinically involved in the pathogenesis of tubotympanic CSOM.

Unlike the previous study by Nuytinck et al. that stated there was a significant relationship between low MBL levels and recurrent acute otitis media [9], this study did not support the assumption that MBL has a relationship with tubotympanic CSOM. There are some issues to consider. First, there has not been any epidemiological studies on MBL levels in Indonesia, including the number/percentage of people with MBL deficiency. The absence of data on normal serum MBL levels in Indonesia has resulted in the criteria of MBL deficiency in Indonesia, which still uses the normal standard for Caucasians, which may differ from Melanesian, especially the Javanese ethnicity. Second, the previous study used child subjects while this study used adult subjects. In children, antibodies from the mother have decreased while adaptive immune system is immature, leading to a weak immune system period [10]. Third, this may be due to the possibility that MBL is not the only component in innate immune systems that play a role in the introduction of pathogenic molecular patterns. MBL works as opsonin and initiates lectin complement activation pathway [20]. In addition, the ability of MBL to identify and bind to bacteria may be involved in the pathogenesis of CSOM [21]. In vitro studies conducted by Garred et al. in 1999 found that MBL has a weak binding with* Pseudomonas aeruginosa*, which is the most common bacterial cause of tubotympanic CSOM [4], and, therefore, opsonization of* Pseudomonas aeruginosa* mediated by MBL cannot occur in in vivo conditions [22].

To our knowledge, this is the first study to specifically examine serum levels of MBL in tubotympanic CSOM patients compared with healthy subjects. Further research is needed with a larger number of samples and to study the relationship between MBL levels in the middle ear with CSOM.

## 5. Conclusion

The findings of the current study indicate that having deficient of MBL levels is not a major factor in the pathogenesis of tubotympanic CSOM. However, the presence of subjects with MBL deficiency in the tubotympanic CSOM group might be considered as playing a role in the tubotympanic CSOM.

## Figures and Tables

**Table 1:** Distribution of age, gender, and MBL level of the study.

Variable	Tubotympanic CSOM *n* = 36	Control *n* = 36	*p*
Age			
Min	17	17	
Max	60	52	
Mean	37.29	30.42	
Gender			
Male	11 (30.5%)	17 (47.2%)	
Female	25 (69.5%)	19 (52.8%)	
Site of infection			
Unilateral	25 (69.4%)		
Bilateral	11 (30.6%)		
Type of CSOM			
Active	34 (72.3%)		
Inactive	13 (27.7%)		
MBL level (ng/ml)			
Min	0.001	188.71	
Max	690.24	794.54	
Mean	354.88	376.27	0.211
MBL level < 100 ng/ml	8 (22.2%)	0 (0%)	

## References

[1] Kong K., Coates H. L. C. (2009). Natural history, definitions, risk factors and burden of otitis media. *Medical Journal of Australia*.

[2] Shenoi P. M. (1988). Management of chronic suppurative otitis media. *Scotts Brown textbook of Otorhinolaryngology*.

[3] Buzi A., Gluth M. B., Black B., Dornhoffer J. L., Gluth M. B. (2016). Chronic ear disease in the modern era: Evolution of treatment, epidemiology, and classification. *The Chronic Ear*.

[4] World Health Organization (2004). *Chronic suppurative otitis media. Burden of illness and management options*.

[5] Monasta L., Ronfani L., Marchetti F. (2012). Burden of disease caused by otitis media: systematic review and global estimates. *PLoS ONE*.

[6] Qureishi A., Lee Y., Belfield K., Birchall J. P., Daniel M. (2014). Update on otitis media - Prevention and treatment. *Infection and Drug Resistance*.

[7] van der Veen E. L., Schilder A. G. M., van Heerbeek N., Verhoeff M., Zielhuis G. A., Rovers M. M. (2006). Predictors of chronic suppurative otitis media in children. *JAMA Otolaryngology - Head and Neck Surgery*.

[8] Juhn S. K., Jung M.-K., Hoffman M. D. (2008). The role of inflammatory mediators in the pathogenesis of otitis media and sequelae. *Clinical and Experimental Otorhinolaryngology*.

[9] Nuytinck L., De Meester E., Van Thielen M., Govaerts P. (2006). Role of mannose-binding lectin (MBL2) genotyping in predicting the risk of recurrent otitis media (rOM). *Advances in Experimental Medicine and Biology*.

[10] Wiertsema S. P., Herpers B. L., Veenhoven R. H. (2006). Functional polymorphisms in the mannan-binding lectin 2 gene: Effect on MBL levels and otitis media. *The Journal of Allergy and Clinical Immunology*.

[11] Mittal R., Kodiyan J., Gerring R. (2014). Role of innate immunity in the pathogenesis of otitis media. *International Journal of Infectious Diseases*.

[12] Lane A. P. (2009). The role of innate immunity in the pathogenesis of chronic rhinosinusitis. *Current Allergy and Asthma Reports*.

[13] Koturoglu G., Onay H., Midilli R. (2007). Evidence of an association between mannose binding lectin codon 54 polymorphism and adenoidectomy and/or tonsillectomy in children. *International Journal of Pediatric Otorhinolaryngology*.

[14] Eren E., Midilli R., Karaca E., Onay H., Karci B., Özkinay C. (2012). Does mannose-binding lectin have a role in adult turkish patients with nasal polyposis?. *Otolaryngology - Head and Neck Surgery*.

[15] The Indonesian Association of Otorhinolaryngology (PERHATI) (2015). Panduan praktik klinik. Panduan praktik klinik prosedur tindakan. *Clinical Pathways*.

[16] Eagan T. M., Aukrust P., Bakke P. S. (2010). Systemic mannose-binding lectin is not associated with chronic obstructive pulmonary disease. *Respiratory Medicine*.

[17] Homøe P., Madsen H. O., Sandvej K., Koch A., Garred P. (1999). Lack of association between mannose-binding lectin, acute otitis media and early Epstein-Barr virus infection among children in Greenland. *Scandinavian Journal of Infectious Disease*.

[18] Alves Pedroso M. L., Boldt A. B. W., Pereira-Ferrari L. (2008). Mannan-binding lectin MBL2 gene polymorphism in chronic hepatitis C: Association with the severity of liver fibrosis and response to interferon therapy. *Clinical and Experimental Immunology*.

[19] Tsutsumi A., Kobayashi T., Ito S. (2009). Mannose binding lectin gene polymorphism and the severity of chronic periodontitis. *Japanese Journal of Clinical Immunology*.

[20] Takahashi K., Chang W.-C., Takahashi M. (2011). Mannose-binding lectin and its associated proteases (MASPs) mediate coagulation and its deficiency is a risk factor in developing complications from infection, including disseminated intravascular coagulation. *Immunobiology*.

[21] Garred P., Brygge K., Sorensen C. H., Madsen H. O., Thiel S., Svejgaard A. (1993). Mannan-binding protein - Levels in plasma and upper-airways and frequency of genotypes in children with recurrence of otitis media. *Clinical and Experimental Immunology*.

[22] Garred P., Pressler T., Madsen H. O. (1999). Association of mannose-binding lectin gene heterogeneity with severity of lung disease and survival in cystic fibrosis. *The Journal of Clinical Investigation*.

